# Improved methods for the detection and quantification of SARS-CoV-2 RNA in wastewater

**DOI:** 10.1038/s41598-022-11187-8

**Published:** 2022-05-03

**Authors:** Beatriz Peinado, Lorena Martínez-García, Francisco Martínez, Leonor Nozal, Maria Blanca Sánchez

**Affiliations:** 1 IMDEA Water Institute, Science and Technology Campus of the University of Alcalá, Avenida Punto Com 2, 28805 Alcalá de Henares, Spain; 2 Center of Applied Chemistry and Biotechnology (CQAB), University of Alcala and General Foundation of Alcala University (FGUA), A-II km 33.600, 28805 Alcalá de Henares, Madrid Spain

**Keywords:** SARS-CoV-2, Microbiology techniques, Water microbiology

## Abstract

Since the start of the COVID-19 pandemic, different methods have been used to detect the presence of genetic material of SARS-CoV-2 in wastewater. The use of wastewater for SARS-CoV-2 RNA detection and quantification showed different problems, associated to the complexity of the matrix and the lack of standard methods used to analyze the presence of an enveloped virus, such as coronavirus. Different strategies for the concentration process were selected to carry out the detection and quantification of SARS-CoV-2 RNA in wastewater: (a) aluminum hydroxide adsorption–precipitation, (b) pre-treatment with glycine buffer and precipitation with polyethylene-glycol (PEG) and (c) ultrafiltration (Centricon). Our results showed that the reduction of organic matter, using the pre-treatment with glycine buffer before the concentration with Centricon or aluminum hydroxide adsorption–precipitation, improved the recovery percentage of the control virus, Mengovirus (MgV) (8.37% ± 5.88 n = 43; 6.97% ± 6.51 n = 20, respectively), and the detection of SARS-CoV-2 in comparison with the same methodology without a pre-treatment. For the concentration with Centricon, the use of 100 mL of wastewater, instead of 200 mL, increased the MgV recovery, and allowed a positive detection of SARS-CoV-2 with N1 and N2 targets. The quantity of SARS-CoV-2 RNA detected in wastewater did not show a direct correlation with the number of confirmed cases, but the study of its upwards or downwards trend over time enabled the detection of an increase of epidemiological data produced in September 2020, January 2021 and April 2021.

## Introduction

The appearance of a new virus, the coronavirus SARS-CoV-2 (Severe Acute Respiratory Syndrome-related coronavirus), responsible for the disease known as COVID-19, has generated a World health crisis. This disease led to the World Health Organization (WHO) declaring a global pandemic on March 11, 2020^1^.

Infectious diseases are a threat to global public health, as the current COVID-19 pandemic has shown. Different techniques are used for the surveillance of infectious diseases, including wastewater analysis^2^. In fact, Wastewater-Based Epidemiology (WBE) is based on the detection of chemical and/or biological compounds in wastewater, which could provide information on human health. WBE has been used for the detection in wastewater of several viruses which cause gastroenteritis before people were diagnosed^3^. It was also used in different countries for the environmental surveillance of polio, being possible to assess the circulation of the virus within the population^4–6^. The use of wastewater provides some advantages, such as analyzing a large population, including symptomatic and asymptomatic individuals. However, it also presents some disadvantages, as wastewater is a complex matrix where the compounds to be analyzed are accompanied by pollutants and will be found diluted^7^.

SARS-CoV-2, responsible for the COVID-19 disease, belongs to the coronaviridae family, viruses with enveloped and single-stranded positive-sense RNA genome^8,9^. The main symptoms of COVID-19 include fever, coughing, dyspnea, sore throat and headaches and some patients also show gastrointestinal symptoms, such as diarrhea (11 ± 2%), vomit and nausea (12 ± 3%)^10^, although recent reports have implied that significant proportions of infected individuals (17.9–30.8%) are asymptomatic^11,12^. Previously, another human coronavirus (HCoV) was detected in the faeces of infected patients^13,14^. In fact, this new coronavirus has also been found in the faeces of infected patients, both with and without diarrhea symptoms^15^, even after respiratory tract tests become negative^16^, as well as in the faeces of asymptomatic people^17^. Viral load in faeces is variable and depends on the course of the infections. Some studies estimated that the amount of the virus that reaches wastewater treatment plants (WWTPs) ranges from 5 × 10^3^ to 10^7.6^ copies/mL in faeces and between 2 copies/100 mL to 3 × 10^3^ copies/mL, due to their dilution in wastewater^16^.

Since the beginning of the pandemic, scientists around the World are using the detection and quantification of SARS-CoV-2 RNA in wastewater to help governments become aware of epidemic outbreaks, allowing them to take appropriate action before its spread. The detection of SARS-CoV-2 in wastewater began in the Netherlands, USA, France, Italy and Australia^18–24^ but more countries, including Spain, now use this analysis to obtain maximum data^25,26^. In Madrid (Spain), a surveillance system with 289 points has been created for the detection of SARS-CoV-2 RNA in wastewater (https://www.canaldeisabelsegunda.es/sistema-vigia) to help health authorities make decisions.

There is already a lot of information available, despite the short time since the virus emergence. During this period, different methods for concentration, extraction kits and the detection of RNA have been described^27,28^. However, not all studies provide information on the efficiency of recovery, the use of controls^1,29^, or how other factors could affect the detection and quantification of the virus, such as storage temperature^30,31^, dilution by rainfall or sampling variability (grab vs. composite, volume).

The aim of this study was to improve different methods of concentration, reducing the presence of organic matter in wastewater by introducing a cleaning step prior to the concentration of the sample, to increase the efficiency of virus recovery. Then, these improved methods were used to follow the presence of SARS-CoV-2 RNA in wastewater and their possible relation to the number of confirmed cases in the population served by the WWTP analyzed in this study.

## Results

### Optimization of sample concentration

One important point during the study of the presence of SARS-CoV-2 RNA in wastewater is the sample concentration method used. After a bibliographic review of the different concentration methodologies described, three of them were chosen (see “Methods”) for a first screening, to select the method to be ultimately used. Samples were spiked with Mengovirus (MgV)^26^ before the concentration, and the recovery percentage was analyzed using the three selected methods, in samples collected from June to July 2020. After several analyses, two of them, method 1 (aluminum hydroxide adsorption–precipitation) and method 2 (treatment with glycine buffer and precipitation with polyethylene-glycol (PEG)) were discarded from further analysis. In the first case, the recovery percentage was very low, close to 1%. In the second case, although the virus recovery was slightly better, this method took too long as it included an overnight incubation. Method 3, ultrafiltration with Centricon, showed a higher recovery percentage (between 2.58 to 6.41%, n = 3) than method 1 and took less time than method 2. For these reasons, method 3 was selected to be used. Nevertheless, the use of Centricon showed an additional problem, as the filter could collapse due to the organic matter present in wastewater. This limited the volume from the sample that could be analyzed to 100 mL, compared to other methods which could be 200 mL or more. To increase the volume that could be analyzed, the possibility of removing more organic matter using a pre-treatment was studied. During the study of the PEG method, it was observed that the treatment with glycine buffer before centrifugation at high speed allowed for the obtention of a supernatant clearer than with a simple centrifugation, used in most of the methods described. This buffer has been described to help detach the virus from organic matter, when it is added over samples or pellets^28,32^. The MgV recovery was analyzed using 100 and 200 mL volumes from the samples, including a first step (pre-treatment with glycine buffer) before proceeding to concentrate with Centricon. In both cases, an increase of MgV recovery, higher to 18% (n = 3) and 13% (n = 2), respectively, was observed. It was decided to include the pre-treatment step before the use of Centricon for later studies (method 4).

### Effect of volume from the sample in the recovery and detection of SARS-CoV-2

The presence of low concentration of SARS-CoV-2 in wastewater suggests that the concentration of a high volume from the sample could improve the detection and quantification. In the reviewed bibliography^18,20,25,26^, different authors use diverse volumes of wastewater to analyze the presence of the virus, mainly between 100 to 200 mL, and even up to 800 mL. However, our previous data suggested that the recovery efficiency of MgV could be optimized when low volumes were used (see above), as described previously using other methodologies^28^. Using method 4, the effect of volumes from the sample (50, 100 and 200 mL) over the recovery efficiency of MgV and detection and quantification of SARS-CoV-2 RNA was analyzed. Samples collected during October and November 2020 were used. A higher level of recovery of MgV was observed when low volumes (50 and 100 mL) rather than high volumes (200 mL) were used (Table 1). The detection and quantification of SARS-CoV-2 RNA were also affected by the volume used from the sample. When 200 mL were used for concentration, the result was not detected for the N2 target (Table 1).

**Table 1:** Concentration of SARS-CoV-2 RNA and percentage of MgV recovery (%) using different sample volumes for concentration.

Sample^a^	Volume (mL)	N1 (log_10_ gc/L)	N2 (log_10_ gc/L)	MgV (%)
1	200	Positive^b^	Positive^b^	5.48
2	200	3.52	N.D.	5.01
3	200	4.12	N.D.	1.94
4	200	N.D.	N.D.	6.04
1	100	3.56	3.53	6.86
2	100	5.09	5.02	25.11
3	100	5.27	5.26	13.12
4	100	4.88	5.14	13.27
1	50	3.70	3.65	13.18
2	50	5.35	5.30	37.58

*N.D.* Not detected.

^a^Samples collected in October (1 and 2) and in November (3 and 4).

^b^Ct < 40 but outside of control curve.

**Figure 1:**
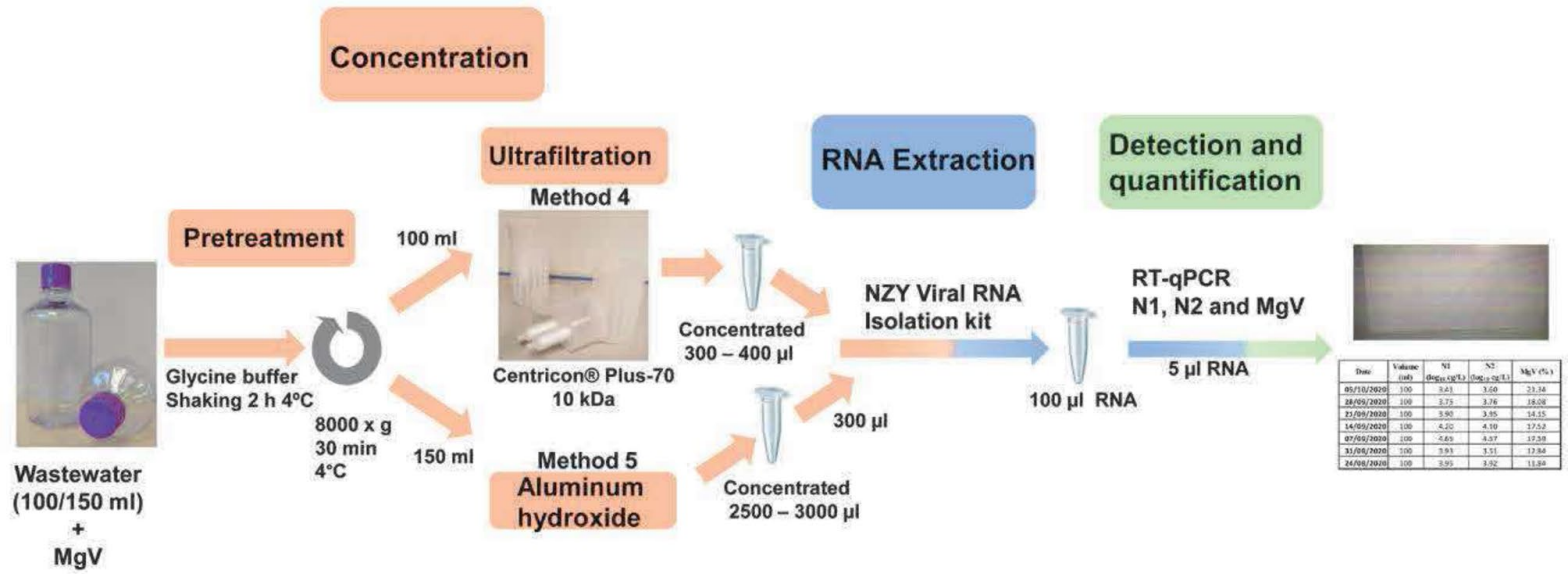
Schematic representation of the experimental procedure proposed to improve methods (method 4 and 5) for the detection and quantification of SARS-CoV-2 RNA in wastewater samples.

For the following analysis, 100 mL from the sample and method 4 for concentration were used for the detection and quantification of SARS-CoV-2 (Fig. 1).

### Detection of SARS-CoV-2 RNA in wastewater samples

Wastewater samples used for the optimization of the concentration procedure (from June to October 2020) were reanalyzed following the new parameters (100 mL from the sample and method 4). The result of comparing different volumes from the same samples using method 4 showed a significant change (P < 0.05), with an increase of MgV recovery percentage when 100 mL were used (Table 2); they were also positive for the presence of virus RNA, with the exception of samples from June and July. While the first analysis showed a difference in quantity between N1 and N2 targets (mean log gc/L N1/N2 1.25 ± 0.27), new data showed great similarity between the two targets analyzed (mean log gc/L N1/N2 1 ± 0.06) (Table 2). A decrease in the amounts of RNA detected in the reanalyzed samples was observed, being lower, in some cases, despite the virus recovery being higher (Table 2). This reduction could be due to frozen samples being used for the reanalysis.

**Table 2:** Concentration of SARS-CoV-2 RNA and percentage of MgV recovery (%) from different sample volumes (200 mL from fresh samples and 100 mL from frozen samples) using method 4.

Date^a^	Volume (mL)	N1 (log_10_ gc/L)	N2 (log_10_ gc/L)	MgV (%)	Volume (mL)	N1 (log_10_ gc/L)	N2 (log_10_ gc/L)	MgV (%)
12/10/2020	200^b^	–	–	0.94	100	4.25	4.60	5.74
05/10/2020	200	Positive^c^	Positive^c^	5.48	100	3.41	3.60	21.34
28/09/2020	200	4.74	4.01	11.74	100	3.75	3.76	18.08
21/09/2020	200	3.55	N.D.	5.91	100	3.90	3.95	14.15
14/09/2020	200	4.50	Positive^c^	6.22	100	4.20	4.10	17.52
07/09/2020	200	4.70	3.15	8.41	100	4.65	4.57	17.59
31/08/2020	200	4.51	4.38	3.33	100	3.93	3.51	12.84
24/08/2020	200	5.05	4.88	14.17	100	3.95	3.92	11.84

*N.D.* Not detected.

^a^Date corresponding to the 24-h composite sample.

^b^Data not suitable, MgV recovery < 1%.

^c^Ct < 40 but outside of control curve.

**Figure 2:**
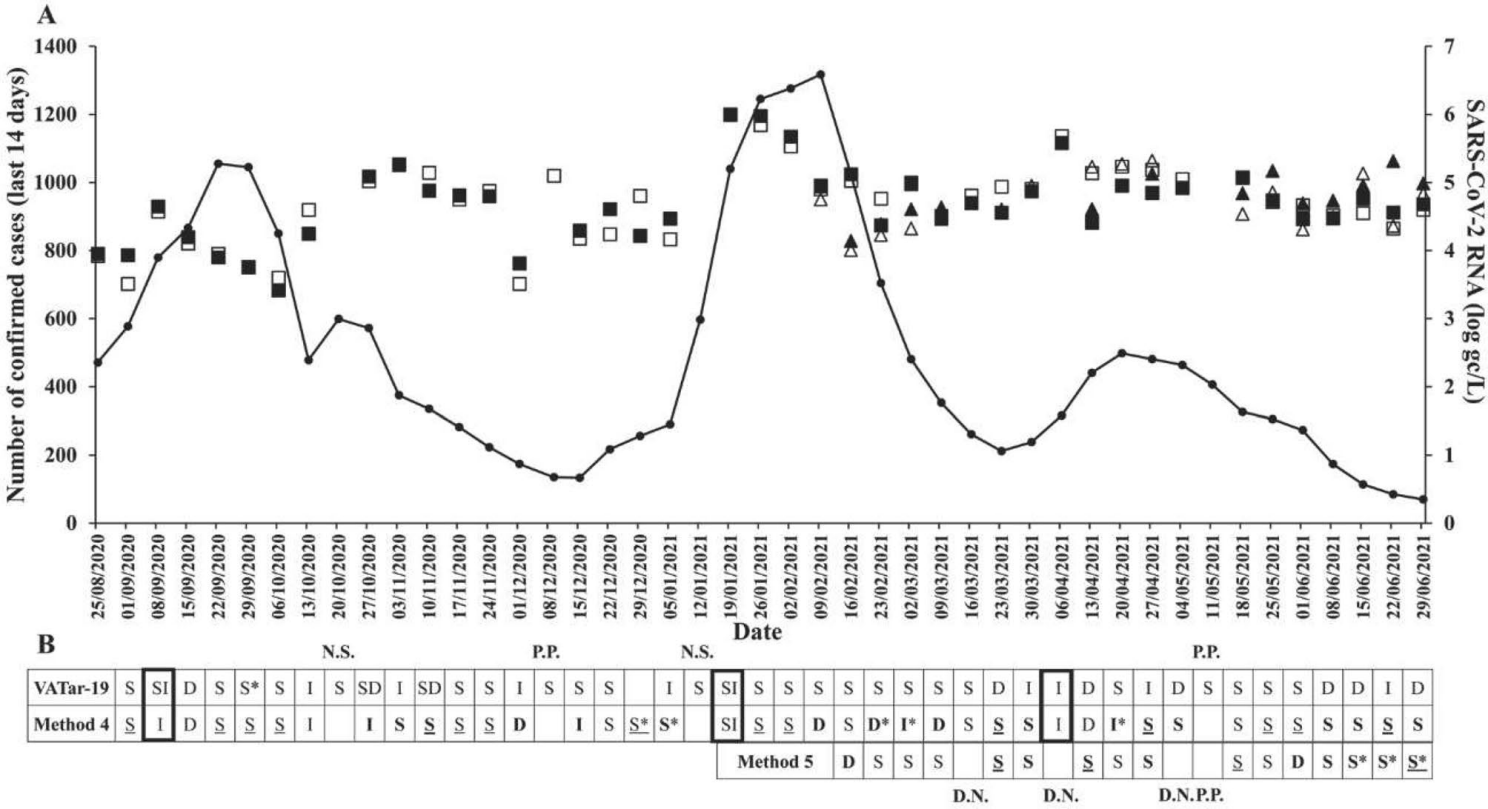
Evolution in the number of cases and quantification of SARS-CoV-2. Panel (**A**) shows the number of cases confirmed in the previous 14 days, and the concentration of SARS-CoV-2 RNA (log gc/L). Data obtained with method 4 is represented by black (N1) and white (N2) squares. Data obtained with method 5 is represented by black (N1) and white (N2) triangles. The date corresponds to the epidemiological data, the day after the wastewater was collected. Panel (**B**) shows the data variation in N1 and N2 targets between weeks obtained in this work with two different methods (4 and 5) and provided by VATar-COVID-19 project. In bold results which vary between the project VATar-COVID-19 and those obtained in this work. Significant decrease (SD) < − 1; Decrease (D) − 1 to − 0.4; Negative Stable (S) − 0.4 to 0; Positive Stable (S) 0 to 0.4; Increase (I) 0.4 to 1 and Significant increase (SI) > 1. *Discrepancy between N1 and N2, data corresponding to N1 trend. *N.S.* Not sample, *P.P.* Presumptive positive (one positive target, two not detected targets), *D.N.* Data not suitable, MgV recovery < 1%.

Wastewater samples collected since October 2020 to June 2021 were analyzed, following the parameters established (100 mL and method 4, Fig. 1), to detect the presence and quantity of SARS-CoV-2 RNA. Data showed a presence of the virus in all analyzed samples (Table S1 and Fig. 2). There were two exceptions: the samples from 08/12/20 and 10/05/2021. In both cases, only one target (N1 or N2) was positive, therefore target E was also analyzed, which was negative. As described below (“Methods”), both samples were defined as presumptive positives^26^.

### Usage of a pre-treatment before the concentration of samples: improving other methods

The use of Centricon could limit the application of this methodology, due to the cost and availability of this consumable, making it inaccessible to some laboratories.

The aluminum hydroxide adsorption–precipitation method (method 1) was discarded in the first study, due to a low MgV recovery. However, this is cheap and not time-consuming, in comparison with the use of PEG (method 2). Our previous data showed a clear increase and better recovery of the control virus when pre-treatment was undertaken, in comparison to when this was not performed. For this reason, the effect on the MgV recovery percentage from adding the pre-treatment step to the aluminum hydroxide adsorption–precipitation methodology was analyzed (method 5, Fig. 1).

Results showed that the addition of a pre-treatment step did not significantly affect (P > 0.05) the recovery of MgV, although it improved the detection and quantification of N1 and N2 targets of SARS-CoV-2 (Table 3), also increasing the number of suitable assays (MgV > 1%).

**Table 3:** Concentration of SARS-CoV-2 RNA and percentage of MgV recovery (%), using the aluminum hydroxide adsorption–precipitation concentration procedure with and without pre-treatment.

Date	Method^a^	Volume (mL)	N1 (log_10_ gc/L)	N2 (log_10_ gc/L)	MgV recovery (%)^b^
08/03/2021	1	200	N.D.	N.D.	1.20
	5	150	4.62	Positive^c^	6.24
01/03/2021	1	200	Positive^c^	Positive^c^	7.88
	5	150	4.61	4.26	14.08
22/02/2021	1	200	–^b^	–^b^	0.31
	5	150	4.39	4.23	4.45
15/02/2021	1	200	–^b^	–^b^	0.45
	5	150	4.14	4.00	1.36
08/02/2021	1	200	3.93	3.78	1.17
	5	150	4.94	4.75	17.55
18/01/2021	1	200	5.73	5.73	1.30
	5	150	6.30	6.16	4.17

^a^Method 1, aluminum hydroxide adsorption–precipitation, method 5, pre-treatment with glycine buffer followed by aluminum hydroxide adsorption–precipitation.

^b^Data not suitable, MgV recovery < 1%.

^c^Ct < 40 but outside of control curve.

*N.D.* Not detected.

The results from the comparison of the two methods with a pre-treatment (method 4 and 5) showed similar results, without significant differences (P > 0.05). However, the use of method 5 didn’t always provide a good control virus recovery (MgV > 1%), when compared to the use of method 4 (Table 4 and Fig. 2).

**Table 4:** Concentration of SARS-CoV-2 RNA and percentage of MgV recovery (%) in samples with a pre-treatment before concentration with Centricon (100 mL and method 4) and aluminum hydroxide adsorption–precipitation (150 mL and method 5).

Date^a^	Method 4			Method 5		
	N1 (log_10_ gc/L)	N2 (log_10_ gc/L)	MgV recovery (%)	N1 (log_10_ gc/L)	N2 (log_10_ gc/L)	MgV recovery (%)^b^
28/06/2021	4.68	4.60	5.91	4.98	4.83	2.78
22/06/2021	4.56	4.32	5.68	5.31	4.36	15.05
14/06/2021	4.76	4.55	6.35	4.96	5.13	7.83
07/06/2021	4.47	4.52	8.00	4.73	4.49	2.43
31/05/2021	4.46	4.67	2.30	4.69	4.31	3.88
24/05/2021	4.74	4.71	6.44	5.17	4.86	2.85
17/05/2021	5.07	5.07	1.88	4.84	4.54	2.24
10/05/2021	Positive^c^	N.D.	12.13	Positive^c^	N.D.	1.99
03/05/2021	4.92	5.05	4.09	–^b^	–^b^	0.06
26/04/2021	4.85	5.18	2.08	5.13	5.32	2.98
19/04/2021	4.95	5.23	2.37	4.95	5.27	1.94
12/04/2021	4.41	5.14	2.43	4.61	5.23	16.32
05/04/2021	5.58	5.68	5.24	–^b^	–^b^	0.83
29/03/2021	4.87	4.91	3.46	4.88	4.95	3.88
22/03/2021	4.56	4.94	2.24	4.57	4.60	23.46
15/03/2021	4.70	4.81	17.88	–^b^	–^b^	0.02
08/03/2021	4.47	4.51	8.33	4.63	Positive	6.24
01/03/2021	5.00	4.97	2.30	4.61	4.32	14.08
22/02/2021	4.37	4.76	6.44	4.39	4.23	4.45
15/02/2021	5.12	5.02	1.88	4.14	4.00	1.36
08/02/2021	4.95	4.90	12.13	4.94	4.75	17.55

^a^Date corresponding to the 24-h composite sample.

^b^Data not suitable, MgV recovery < 1%.

^c^Ct < 40 but outside of control curve.

*N.D.* not detected.

### Epidemiological status and SARS-CoV-2 RNA data

The objective of analyzing the presence of SARS-CoV-2 in wastewater is to use such presence as an early warning, detecting the virus before cases appear, and even following the evolution of the epidemic. SARS-CoV-2 RNA data was compared to the epidemiological situation of the population, corresponding with the WWTP analyzed, obtained from the Network of Epidemiological Surveillance of the Community of Madrid. In this work, the number of confirmed cases over the previous 14 days (last access 07/07/2021) was selected as a parameter to follow the evolution of the pandemic and compared with the quantity of SARS-CoV-2 RNA obtained via methods 4 (100 mL) and 5 (150 mL) (Fig. 2). It was not possible to establish a correlation between the amount of SARS-CoV-2 RNA in wastewater and the epidemiological data (number of cases), observing that the same number of genome copies corresponded to different epidemiological values (number of cases).

Another approach used was to analyze the trend in the presence of the virus, following the conditions described in the project VATar-COVID-19 (see “Methods”). Data obtained via both methods (4 and 5), was compared to data provided by the VATar-COVID-19 project for the same WWTP. As in the VATar-COVID-19 project, data obtained via method 4 allowed us to detect the increase of SARS-CoV-2 RNA in September 2020, January 2021, and April 2021 (Fig. 2B), which corresponded with an increase in the epidemiological data.

The definition used as stable variation (see “Methods”) covers a very wide difference, between − 0.4 and + 0.4. To increase the information about the pandemic trend, it was also considered whether the change was negative (negative stable, from − 0.4 to 0) or positive (positive stable, from 0 to + 0.4), for at least two or more consecutive weeks. This allowed for the detection of decreases of SARS-CoV-2 RNA (from 22/09/20 to 06/10/20, from 10/11/20 to 24/11/20, followed by a decrease; from 26/01/21 to 02/02/21 followed by a decrease and from 25/05/21 to 01/06/21), which agreed with epidemiological data (Fig. 2).

## Discussion

Since the emergence of the COVID-19 pandemic, Science has been attempting to help: from looking for treatments to developing vaccines. Another important aspect in the fight against the SARS-CoV-2 virus has been avoiding its dissemination among people, through the application of different control measures such as the use of masks, lockdowns, or mobility restrictions. For this purpose, the analysis of the presence of SARS-CoV-2 RNA in wastewater has demonstrated to be a successful tool, detecting the presence of the virus even before clinical symptoms appear^18–24,33^.

The Wastewater-Based Epidemiology (WBE) has been previously used for the detection of other viruses, mainly gastroenteric viruses^3–6^. In the case of the new coronavirus, SARS-CoV-2, due to the lack of a clear methodology for its detection and quantification, multiple methodologies have been described in recent bibliographies^20,25–27,29^. Methods described vary in different aspects, such as the volume or type of sample (grab or composite), storage conditions, method of concentration, inclusion of process control, RNA extraction kits or target genes used, among others^1,27,29^.

Most of the methods analyzing the presence of SARS-CoV-2 comprise three steps: concentration, extraction of RNA and detection and quantification of virus. The concentration step is considered important, due the low level of virus found in wastewater and the complexity of the sample, which presents a lot of pollutants that can interfere with the process. However, currently few methods have been described where genetic material was extracted directly from wastewater without a previous concentration^30,34^ or even the presence of virus was determined directly in raw sewage^35^. When this work began, most of the methodologies described the use of a concentration step. Among the different concentration methodologies described in the bibliography, we were interested in a particular one which was able to concentrate samples in a short time, without any requirements for sophisticated equipment, while achieving a high virus recovery and at a low cost and keeping constant other factors, such as the sampling conditions (composite samples), the RNA extraction (NZY Viral RNA Isolation Kit) and the target detected (N1 and N2). A quick screening of the three methods selected allowed us to discard the use of PEG, because it involved at least two days for the concentration of the sample; and initially the use of aluminum hydroxide adsorption–precipitation due to the low recovery of the control virus, which could affect the detection in cases of low presence of the virus. The third alternative, ultrafiltration with Centricon, was selected although that also presented problems, such as the limitation of the volume of sample that could be handled, cost and availability.

The main difficulty associated to the use of Centricon is the volume to be used which correlate with the pollutants present in the wastewater. The presence of organic matter limits the volume to be load, because could cause the filters to collapse. Indeed, Pino et al.^36^ showed differences in virus recovery using wastewater and deionized water, suggesting a negative effect of the presence of pollutants in wastewater analysis. Other studies have demonstrated that the removal of the solid can reduce the virus recovery, since the virus is retained in the pellet^33,37^. Despite that, most of the concentration methods include a first centrifugation to remove suspended solid to avoid interferences. Hence, in this study, other options were examined to reduce the prevalence of organic matter without eliminating the presence of the virus. The addition of glycine buffer before a centrifugation, used on a PEG method, has been described to reduce the organic matter and to detach the virus^32^. The inclusion of this step, which was denominated pre-treatment, do not increase the time of processing but allowed us to concentrate different volumes from samples, without running the risk of collapsing the filter (method 4) and improve the detection of SARS-CoV-2 RNA (Table 2).

Another aspect of sample concentration that is not much known is the effect of volume used on the recovery of control virus and detection and quantification of SARS-CoV-2 RNA. The use of different volumes depending on the method used has been described^1,18,20,25,26^, but few studies show the effect of different volumes with the same concentration method^30^. Data obtained for the analysis of different volumes (50, 100 and 200 mL), with method 4, showed that higher volume processed did not mean a better recovery of the control virus or an increase in the levels of SARS-CoV-2 RNA detected (Table 1). Indeed, the analysis of 100 mL from the sample showed a significant increase (P < 0.05) in the recovery of the control virus and allowed for the quantification of SARS-CoV-2 in all samples, in comparison with a higher volume, 200 mL (Table 2). These results could be associated to the presence of pollutants, higher in larger volumes, which could interfere during the concentration and later extraction of viral RNA, affecting the detection of SARS-CoV-2.

Although Centricon allowed to obtain results in a short time and solved the problem of volume, there was still the issue of its cost and availability. This could mean that not all laboratories would be able to afford this method and, in some situations, as it happened at the beginning of the pandemic, this product may be scarce. So, it could become necessary to use an alternative method. The adsorption–precipitation of aluminum hydroxide is cheap, although was ruled out by a low recovery of the control virus. The addition of a pre-treatment, as in the case of Centricon, improved the results of the detection and quantification of virus SARS- CoV-2 (Table 3), making it (method 5) a good alternative to using Centricon. Despite the improvement in the detection of SARS-CoV-2 RNA, the recovery of the control virus did not show a significant increase, in comparison with the method without pre-treatment, method 1 (Table 3) or other similar studies^26,38^. In general, the use of a pre-treatment seemed to improve SARS-CoV-RNA detections, regardless of the subsequent concentration method (ultrafiltration or adsorption–precipitation), but it was not always related to an improvement in the control virus recovery (Table 4). These data suggests that the use of pre-treatment could be applied to other concentration methods to improve the detection of SARS-CoV-2.

Another factor that must be considered is the proper storage conditions to maintain samples until analysis are completed. It is recommended that wastewater should be analyzed as soon as sample is collected, but this is not always possible. In this study, after collecting wastewater, a fraction of each sample was analyzed for detection and quantification of viral RNA, and the remained sample was stored (frozen), in case of need for reanalysis. In fact, some of them were reanalyzed after the optimization of the concentration (Table 2); in others cases samples were analyzed after 24h of collection or reanalyzed when results came back inconclusive (Table S1). When stored samples were reanalyzed, different results were observed between frozen and fresh samples. The frozen samples showed a better recovery of the control virus, but not always corresponding with a higher level of SARS-CoV-2 RNA, being indeed lower than in fresh samples (Table 2). This could be due to the improvement of the method (pre-treatment of the sample) and the fact that the control virus was not frozen with the samples, unlike SARS-CoV-2. This suggests a possible negative effect of storage on RNA stability. The effect of storage temperature on the detection and quantification of viral RNA has not been examined in depth and further studies will be required to determine how and how much storage may affect the presence of RNA. Our results agree with others showing that freezing seems to reduce the presence of viral RNA in wastewater^30,31^.

The use of a control virus provides important information of how efficient and reliable the recovery of the virus of interest, SARS-CoV-2, is. However, the combination of different control viruses and methods used by other groups, makes difficult to compare recovery efficiencies. The data got in this study showed low recovery and a great variability for both methods, 8.37% ± 5.88 n = 43 (method 4); 6.97% ± 6.51 n = 20 (method 5), in comparison with other data found in the bibliography relating to other control viruses, such as 1.6–2.6% for Murine Norovirus, 26.7–64.7% for MHV, 73 ± 50% for F-specific RNA, 42% and 30% for *Pseudomonas* phage φ6^20,27,36^. Even when results using the same control virus and method were compared, such as virus recovery showed in this work using method 5 (6.97% ± 6.51), data from Randazzo et al.^26^ (10% ± 2.1%) and data from Pérez-Cataluña et al.^38^ (9.0 ± 2.2%), there are other factors which could affect the results, such as the type of sample or the kits used for the RNA extraction and the RT-qPCR quantification.

The aim of detecting and quantitying SARS-CoV-2 RNA in wastewater is to know the state of the pandemic, without analyzing many people. The relation between both data, number of infected people and the number of genomic copies should be linear, but that is not shown by data. There is a high number of unknown factors which affect the presence of virus, such as the weather (dry or rainfall day); the type of sample (grab vs composite, fresh or frozen, volume); the variability of viral load in faeces (depending on the course of the viral load in the population infected); the presence of other compounds in the wastewater, etc. Another factor to consider is the reliability of the epidemiological data. All infected people are not always represent, due to the presence of undiagnosed asymptomatic individuals, people who have recovered from the infection^16^, or the variability in the number of tests performed. Despite not being able to establish a relationship between the epidemiological data and the quantity of SARS-CoV-2 RNA detected in wastewater, information is useful when trend over time is analyzed, as described in the VATar-COVID-19 project. Data showed in this work, concurs with data provided by VATar-COVID-19 for the same WWTP, allowing to detect growth in the presence of SARS-CoV-2 RNA that corresponded to an increase in epidemiological data (September 2020, January 2021, and April 2021) (Fig. 2B), when method 4 was used. These findings agree with other studies which have demonstrated that similar results could be obtained using different methods, and that it is more important to consistently use the same method than the method itself^30^. Yet, the comparison showed some dissimilarities (Fig. 2B), probably due to differences in conditions and method of analysis. The same trend analysis was carried out with the data obtained via method 5. In this case, there were more dissimilarities to the data from the VATar-COVID-19 project, due to the lower amount of data available (from February to June).

The trend study allowed for the detection of an increase in viral RNA epidemiological data, but not a decrease. It tends to take a longer period of time to see a drop in the number of active cases, usually several weeks, while increases occur very quickly (Fig. 2). A negative trend in the presence of viral RNA is not usually detected as a decrease but as stable, probably due to the definition of stable: between − 0.4 to + 0.4, a wide range. However, when this data was divided into two parts [a negative stable (− 0.4 to 0) and a positive stable (0 to 0.4)], and analyzed over at least two or more consecutive weeks, a decrease in viral RNA was also detected (from 22/09/20 to 06/10/20, from 10/11/20 to 24/11/20 followed by a decrease, from 26/01/21 to 02/02/21 followed by a decrease and from 25/05/21 to 01/06/21), in agreement with a decrease in epidemiological data (Fig. 2). We consider that, for a comprehensive study into the evolution of the pandemic, it is important to detect not only increases but also decreases in the presence of SARS-CoV-2 RNA.

In summary, the addition of a pre-treatment, could be applied to increase the detection of SARS-CoV-2 RNA in wastewater. Regardless, there are still several factors, such as storage temperature, variety of sample (grab or composite) or volume from the sample to be analyzed, which require further studies for a better viral RNA detection methodology. Despite these challenges, the detection of SARS-CoV-2 RNA in wastewater by RT-qPCR has demonstrated to be a valuable tool for the detection of increases in cases before they are shown by epidemiological data, while the study of its trend over time has enabled to follow the evolution of the pandemic.

## Methods

### Wastewater sampling

Influent water samples were collected from a wastewater treatment plant (WWTP) in Torrejón de Ardoz, a city located 19 km to the East of Madrid (Spain), with a population of more than 100,000 inhabitants (132,853 in January 2020). A 24 h daily composite wastewater sample (time proportional) was collected in polycarbonate (PC) bottles. One part of the sample was stored at − 20 °C, and the other part was processed in the first 24 h after collection, being kept at 4 °C until processing.

Wastewater samples were collected once a week for one year (51 samples from June 2020 to June 2021), with some exceptions when it was not possible to collect samples.

### Concentration procedure

For the concentration of viruses from wastewater samples, different methodologies previously published were used, as follows.** Method 1** is based on aluminum hydroxide adsorption–precipitation^26^. In brief, 200 mL from the sample were centrifuged for 30 min at 4600×*g* to remove any solids present. Then, before and after the addition of AlCl_3_ 0.9 N (1 part to 100 parts of sample), the pH was adjusted to 6, and the sample was shaken for 15 min at 150 rpm. Viruses were then concentrated by centrifugation during 20 min at 1700×*g* and the pellet was resuspended in 10 mL of beef extract at 3%, then shaken for 10 min at 200 rpm and centrifugated for 30 min at 1900×*g*. The pellet was resuspended in 2 mL of PBS 1 ×, and part of it used for RNA extraction. **Method 2** consists of a treatment with glycine buffer and precipitation with polyethylene-glycol (PEG)^25,32^. In this method, 200 mL from the sample were added to 25 mL of glycine buffer (0.05 M glycine, 3% beef extract, pH 9.5), shaken for 2 h at 4 °C and 300 rpm, and centrifuged for 30 min at 4 °C and 8000×*g* to detach virions bound to organic matter and remove solids present in the sample. This process has been called pre-treatment. Then the pH of recovered supernatant was adjusted to 7.0–7.2 with HCl 5 M. 22.5 g of PEG 6000 and 3.94 g of NaCl were added and the sample was shaken overnight at 4 °C and 300 rpm. The next day, the sample was centrifuged for 90 min at 4 °C and 10,000×*g*. The pellet was resuspended in 1 mL of PBS 1 ×, sonicated, and centrifuged again for 15 min at 3000×*g*. The supernatant was then recovered for RNA extraction. **Method 3** involves the ultrafiltration with Centricon^18,20^. In brief, 100 mL from the sample were first centrifuged for 30 min at 4600×*g* to remove any solids present. The supernatant (100 mL) was then concentrated by filtering successively twice 50 mL in the same Centricon^®^ Plus-70 centrifugal filter with a cut-off of 10 kDa (MerckMillipore) following the manufacturer’s instructions. A volume ranging between 300 to 400 µL was recovered for RNA extraction.

Two other methods were used which resulted from the modification of methods 1 and 3 (see above), developed in this work. In both cases, the first centrifugation to remove any solids present in the wastewater was changed to the pre-treatment with glycine buffer (method 2), followed by the ultrafiltration with Centricon^®^ Plus-70 centrifugal filter with a cut-off of 10 (**method 4**) or by aluminum hydroxide adsorption–precipitation (**method 5**). The volume used was 100 mL for method 4, and 150 mL for method 5.

As control of virus recovery, before proceeding to the concentration (method 1, 2 and 3) or pre-treatment (method 4 and 5), wastewater samples were spiked with 10 µL of a 10^–2^ dilution of Mengovirus (MgV) (CECT 100000). For method 5, samples were spiked with 100 µL of a 10^–2^ dilution of MgV^26^.

Sample concentrates were stored at − 20 °C until RNA extraction.

### RNA extraction and RT-qPCR analysis

Regardless of the methodology used for wastewater concentration, in all cases RNA was extracted using NZY Viral RNA Isolation Kit (MB40701) NZYTech, following the manufacturer’s instructions. RNA was recovered in 100 µL of RNase free water and stored at − 80 °C until analysis.

SARS-CoV-2 RNA was detected and quantified by RT-qPCR using One-step NZY RT-qPCR Probe kit, ROX plus (MB34702) NYZTech in a real time PCR (AB7300) instrument. The detection of SARS-CoV-2 RNA was performed using kit 2019-nCoV RUO Kit (Integrated DNA Technologies, IDT) which contains the oligonucleotides and probes for N1 and N2, two regions of the nucleocapsid gene (N gene) as described by the Centers for Disease Control and Prevention^39^. The program used was at 50 °C for 15 min for reverse transcription, followed by 95 °C for 3 min, and then 45 cycles of 95 °C for 5 s and 55 °C for 30 s. As positive controls, and for quantification (control curve) for N1 and N2 targets, 2019-nCoV_N_Positive Control (Integrated DNA Technologies, IDT) was used. The detection of a second gene, E (envelope gene), was performed using E Assay_First Line Screening (Integrated DNA Technologies, IDT) following the conditions described by Corman et al. ^40^, with the exception of the reverse transcription that was performed at 50 °C for 15 min, following the manufacturer’s instructions for the enzyme used. As positive controls and quantification (control curve) for the E gene, 2019-nCoV_E Positive Control (Integrated DNA Technologies, IDT) was used. For inhibition control, the detection of N1 and N2 targets was performed using 5 µL of undiluted and ten-fold diluted RNA samples. When the difference between both was ≤ 0.5 log, the data was the result of the average, in other cases (≥ 0.5 log) the data corresponded to the diluted sample.

The detection of MgV (CECT 100000) as an internal control in the recovery process was also carried out in triplicate, with 5 µL of RNA following the conditions described by ISO 15216-1^41^, with the exception of the reverse transcription that was performed at 50 °C for 15 min, following the manufacturer’s instructions for the enzyme used. The recovery percentage was determined using a control curve generated with RNA extracted from 10 µL of original MgV. For method 5, the method previously described was followed^26^. Oligonucleotides and probe for MgV detection were provided by Sigma/Merck and Eurofins respectively.

The process was considered suitable when MgV recovery was > 1%. The detection of SARS-CoV-2 was positive when N1 and N2 targets Ct < 40, or when one was not detected and the E target was positive. A sample was considered presumptive positive when only one target (N1 or N2) was detected and the E target was not detected^26,42^. The quantity of SARS-CoV-2 RNA was provided as a logarithm of genomic copy by L (gc/L), when Ct were within the control curve (≥ 10 gc), without the recovery correction.

### Epidemiological and SARS-CoV-2 data collection

Epidemiological data was obtained from the Epidemiological Surveillance Network of the Community of Madrid (https://datos.comunidad.madrid/catalogo/dataset/covid19_tia_muni_y_distritos), which provides a weekly report with data on confirmed cases, confirmed cases with active infections, and cumulative incidence rates for the past 14 days. Data detailing confirmed cases in the last 14 days was used (07/07/2021 last access).

To carry out the studies on the trend of the presence of SARS-CoV-2 in wastewater, data was analyzed following the conditions described in the VATar-COVID-19 project (https://www.miteco.gob.es/es/agua/temas/concesiones-y-autorizaciones/vertidos-de-aguas-residuales/alerta-temprana-covid19/default.aspx), organized by the Ministry for the Ecological Transition and the Demographic Challenge and the Ministry of Health of Spain. In this project, the change in the genetic material (log gc/L) is analyzed week on week, defining five situations: a significant decrease (< − 1), a decrease (− 1 to − 0.4), a stable (− 0.4 to 0.4), an increase (0.4 to 1) and a significant increase (> 1). The quantification of the presence of SARS-CoV-2 is performed following the concentration method described by Randazzo et al. 2020 (https://www.miteco.gob.es/es/agua/temas/concesiones-y-autorizaciones/vertidos-de-aguas-residuales/alerta-temprana-covid19/VATAR-COVID19-Protocolo-deteccion-SARS-CoV-2-aguass.aspx, September 2020 version).

In the cases where it was not possible to obtain a sample, sample positive (P), presumptive positive (P.P.) or not detected (N.D.) data were compared to the previous week when data was available. Additionally, the project provides weekly information on the trend of the presence of SARS-CoV-2 RNA in wastewater in different WWTPs in Spain, including the WWTP studied in this work.

### Statistical analysis

The GNU PSPP program (https://www.gnu.org/software/pspp/get.html) was used for statistical analysis, where P < 0.05 was considered statistically significant.

### Ethics approval

No human or animal studies are presented in this manuscript.

## Supplementary Information

Supplementary Table S1.

## Data Availability

All data generated or analyzed during this study is included in this published article and its supplementary information files.
