# Supplementary material for: Improved methods for the detection and quantification of SARS-CoV-2 RNA in wastewater
Source: Sci Rep. 2022 May 3;12:7201. doi: 10.1038/s41598-022-11187-8 (PMC9063616; doi:10.1038/s41598-022-11187-8)
Supplement: Supplementary file 1 — Supplementary Table S1. [file 41598_2022_11187_MOESM1_ESM.pdf]

Improved methods for the detection and quantification of SARS-CoV-2 RNA in wastewater

Beatriz Peinado [1], Lorena Martínez-García [1], Francisco Martínez [1], Leonor Nozal [2] and Mª Blanca Sánchez [1]*

[1]IMDEA Water Institute, Science and Technology Campus of the University of Alcalá, Avenida Punto Com 2, 28805, Alcalá de Henares, Spain

[2]Center of Applied Chemistry and Biotechnology (CQAB), University of Alcala and General Foundation of Alcala University (FGUA), A-II km 33.600, 28805, Alcalá de Henares, Madrid, Spain.

* Corresponding author: e-mail: blanca.sanchez@imdea.org. Phone (+34) 91 830 59 62. Fax (+34) 91 830 59 61.

Table S1. Concentration of SARS-CoV-2 RNA and MgV recovery percentage (%) in the analyzed sample.

Date[a]	Volume (ml)	N1 (log_{10} gc/L)	N2 (log_{10} gc/L)	E (log_{10} gc/L)[c]	MgV (%)	Sample[d]
28/06/2021	100	4.68	4.60	-	5.91	F
21/06/2021	100	4.56	4.32	-	5.68	F
14/06/2021	100	4.76	4.55	-	6.35	F
07/06/2021	100	4.47	4.52	-	8.00	F
31/05/2021	100	4.46	4.67	-	2.30	F
24/05/2021	100	4.74	4.71	-	6.44	F
17/05/2021	100	5.07	5.07	-	1.88	F
10/05/2021	100	Positive[b]	N.D.	N.D.	12.13	S
03/05/2021	100	4.92	5.05	-	4.09	F
26/04/2021	100	4.85	5.18	-	2.08	F
19/04/2021	100	4.95	5.23	-	2.37	F
12/04/2021	100	4.41	5.14	-	2.43	F
05/04/2021	100	5.58	5.68	-	5.24	F
29/03/2021	100	4.87	4.91	-	3.46	F
22/03/2021	100	4.56	4.94	-	2.24	F
15/03/2021	100	4.70	4.81	-	17.88	F
08/03/2021	100	4.47	4.51	-	8.33	F
01/03/2021	100	5.00	4.97	-	10.51	F
22/02/2021	100	4.37	4.76	-	3.52	F
15/02/2021	100	5.12	5.02	-	9.32	F
08/02/2021	100	4.95	4.90	-	3.54	F
01/02/2021	100	5.67	5.53	-	6.55	F
25/01/2021	100	5.98	5.84	-	7.97	F
18/01/2021	100	6.00	5.99	-	2.81	F
04/01/2021	100	4.47	4.16	-	4.00	S
28/12/2020	100	4.22	4.80	-	5.87	S
21/12/2020	100	4.61	4.24	-	7.17	S
14/12/2020	100	4.29	4.17	-	3.15	S
08/12/2020	100	N.D.	5.10	N.D.	5.97	S
30/11/2020	100	4.31	5.14	-	6.08	S
23/11/2020	100	4.80	4.88	-	2.51	F
16/11/2020	100	4.81	4.75	-	13.69	F
09/11/2020	100	4.88	5.14	-	13.27	F
02/11/2020	100	5.27	5.26	-	13.12	F
26/10/2020	100	5.09	5.02	-	25.11	F

[a] Date corresponding to the 24-hour composite sample

[b] Ct < 40 but outside of control curve

[c] Data provide only when N1 or N2 were not detected.

[d] Sample was analyzed after collection, fresh (F) or after being frozen, stored (S).

N.D. Not detected
